# Prevalence of food allergy and its association with atopic dermatitis in Iran: Results from the PERSIAN birth cohort

**DOI:** 10.1016/j.jacig.2024.100385

**Published:** 2024-12-10

**Authors:** Kylie Jungles, Maryam Sharafkhah, Keerthi Bansal, Marjan Moallemian Isfahani, Nashmia Qamar, Sareh Eghtesad, Roya Kelishadi, Navid Danaei, Amir Houshang Mehrparvar, Hamid Hakimi, Hossein Poustchi, Mahboobeh Mahdavinia

**Affiliations:** a Division of Allergy and Immunology, Department of Medicine and Department of Pediatrics, Rush University Medical Center, Chicago, Ill; b Liver and Pancreatobiliary Diseases Research Center, Digestive Diseases Research Institute, Tehran University of Medical Sciences, Tehran, Iran; c Division of Allergy and Immunology, Ann & Robert H. Lurie Children’s Hospital of Chicago, Northwestern University Feinberg School of Medicine, Chicago, Ill; d Child Growth and Development Research Center, Research Institute for Primordial Prevention of Non-Communicable Disease, Isfahan University of Medical Sciences, Isfahan, Iran; e Department of Pediatrics, School of Medicine, Semnan University of Medical Sciences, Semnan, Iran; f Industrial Diseases Research Center, Shahid Sadoughi University of Medical Sciences, Yazd, Iran; g Immunology of Infectious Diseases Research Center, Rafsanjan University of Medical Sciences, Rafsanjan, Iran; h Division of Allergy and Immunology, Department of Medicine and Department of Pediatrics, UT Health Houston, Houston, Tex

**Keywords:** Food allergy, atopic dermatitis, cohort

## Abstract

**Background:**

The incidence of food allergy (FA) has been increasing worldwide, causing growing concern on a global scale.

**Objective:**

This birth cohort study analyzes the incidence of reported FA and other atopic comorbidities in children from birth to age 2 years who were living in 4 urban and semiurban areas in Iran.

**Methods:**

Children were followed from birth until age 24 months, with follow-up questionnaires administered through parent or guardian interviews conducted when the children were aged 2, 4, 6, 12, and 24 months.

**Results:**

The rate of physician-diagnosed FA reported by parents or guardians was higher than expected, with a cumulative incidence of 7.7% in children younger than 24 months. The highest prevalence of FA was found in Yazd, the most urban of the 4 cities studied. Breast-feeding was associated with a decreased cumulative risk of FA at age 12 months, with only 5% of breast-fed children developing parent-reported pediatrician-diagnosed FA compared with 13% of infants who never received breast milk after birth.

**Conclusion:**

This study provides valuable insight into the incidence of FA in the Middle East, which has previously not been reported on, and it is crucial in our understanding of global FA prevalence. The study demonstrates a high incidence of FA in an area with historically lower rates and confirms that breast-feeding does prevent FA during infancy in this population.

Food allergy (FA) is an increasing public health concern and an important part of the “second wave” of the allergy epidemic, following asthma.^1^ The increasing incidence of FA worldwide over the past few decades has raised tremendous concern among patients and clinicians alike, particularly given the impact on quality of life of both patients and families, as well as economic implications.^2^ Although FA was initially thought to primarily affect industrialized and Western nations, we are currently seeing a paradigm shift in increasing incidence of FA globally, affecting all countries worldwide.^2^^,^^3^ In some Western countries such as the United States, IgE-mediated FA affects approximately 3% to 8% of children.^2^^,^^4^ The disease burden in many other nations is more difficult to estimate, given the paucity of reported population-based data; however, current estimates suggest that significant differences in FA prevalence in different regions of the world remain.^2^^,^^3^ Several factors that may help to explain these observed differences have been hypothesized to contribute to the development of FA; these factors include but are not limited to genetic, environmental, behavioral, and cultural factors. Some studies have suggested that the difference in FA incidence in economically emerging nations could be attributed to a closer relationship with the natural environment (a close relationship to agriculture, farming, and livestock), as well as to dietary differences, which may lead to protective effects on the gut microbiome.^5^ Additional research into these factors, particularly in areas of the world experiencing a lower incidence of FA, can provide important insights into identifying potential risk factors as well as protective factors in the development of FA.

Determining the accurate global incidence and prevalence of FA has remained a challenge owing to varying study methodologies, varying ages of enrolled children, and the cross-sectional nature of most reports. Furthermore, discrepancies exist in capturing accurate data, as most available data on FA prevalence are based on self-reported patient surveys rather than clinician-diagnosed FA. Additionally, some studies rely solely on the use of various IgE FA assays (skin testing and serologic testing), which indicate food sensitization but are not necessarily diagnostic of clinical FA without a supportive clinical history.^6^^,^^7^ The most accurate methodology to measure the prevalence of a childhood condition such as FA is with a prospective birth cohort. This methodology has been imperative in identifying risk factors during the initial stages of life and association with other atopic conditions. It also provides valuable information on the time course of allergen sensitization in early life, which can identify inciting risk factors to help determine preventive measures in the development of FA. Although there have been birth cohort studies in most industrialized nations, these data are not currently available for many areas, such as the Middle East.^8^ Many Middle Eastern countries such as Iran have undergone rapid urbanization over the past few decades, making them ideal populations to study when considering how urbanization can affect FA and atopic disease. We report a novel study of the incidence of FA in the Middle East obtained from a birth cohort through which we sought to understand the risk factors for development of FA in this population.

## Methods

The Prospective Epidemiologic Research Studies (PERSIAN) birth cohort is a multicenter comprehensive study, aimed at studying developmental origins of health and diseases in the Iranian population.^9^ The PERSIAN birth cohort study aimed to recruit 12,000 participants over 10 years (for more details on the PERSIAN birth cohort, please see www.persian-bc.family). The data for this article are based on an embedded smaller cohort intended to assess the reported incidence of atopic conditions such as FA, wheezing, allergic rhinitis, and atopic dermatitis (AD). This nested cohort participants were recruited over a 2-year period (September 2018-December 2020). This study aimed to investigate the incidence of FA and other atopic comorbidities in children living in 4 urban and semiurban areas in Iran. All pregnant mothers visiting selected maternal clinics across these cities regardless of any risks for atopic diseases were invited to participate. Those who agreed and signed an informed consent form were enrolled. Their children were then followed until age 24 months with follow-up questionnaires administered by in-person interviews with parents or guardians when the children were 2, 4, 6, 12, and 24 months of age. Data on late pregnancy events, mode of delivery, and breast-feeding history were captured for all participants. All sites had a universal preplanned protocol that was fully followed; each site had coordinators who were trained and observed by a single group of study trainers, who frequently traveled to all 4 sites to avoid site variations. The study trainers and site principal investigators met with the study coordinators, who were in charge of recruitment and interviews biannually. The capture and storage of data were monitored during these scheduled meetings, and any raised issues and difficulties were addressed. Study staff were also educated about atopic conditions, as well as FA, AD, and wheezing and ways in which these conditions differ from other types of food intolerance or skin eruptions.

FA was defined as a rapid-onset adverse response to a food with symptoms that may involve different systems, including the skin, gastrointestinal tract, respiratory tract, and cardiovascular system. The diagnosis of FA in the study participants was captured through surveys and confirmed by a trained study coordinator through an interview-based survey. For purposes of this study, it was referred to as parent-reported FA. Furthermore, parents were asked whether the child was diagnosed as having FA by a pediatrician through evaluation and testing; report of IgE-mediated symptoms; and response to treatment with antihistamines, steroids, or epinephrine. Type of food, reaction history, and age at first reaction were captured and recorded through this in-person interview by trained study staff. History of skin disease (AD), lower respiratory disease (wheeze and/or asthma), and allergic rhinitis was also obtained.

The family’s history of atopy was also captured; a positive family history was defined as a history of any physician-diagnosed atopic condition (FA, AD, asthma, or allergic rhinitis) in a first-degree relative of the enrolled infant based on questions. These data were captured through questions asking about the history of any of these conditions in all first-degree relatives.

AD was confirmed by questions regarding skin symptoms, chronicity of the symptoms, and family history based on the diagnostic criteria of the American Academy of Dermatology’s Atopic Dermatitis Clinical Guideline (aad.org). The children with AD were grouped on the basis of time of onset and persistence of their AD symptoms. The children were stratified into 4 groups: those who had never had AD, those who had early transient AD, those with early persistent AD, and those with late AD. Those with the 2 “early” phenotypes had onset before age 1 year, and those with transient AD experienced full resolution of symptoms before age 1 year. The “late” phenotype was defined as onset of AD after age 1 year. The children with FA were grouped on the basis of age of first symptoms indicative of an IgE-mediated reaction and means of diagnosis.

The surveys were completed through interviews conducted via paper forms by the research coordinators at the initial recruitment visit, followed by in-person assessments and interviews conducted when the children were 2, 4, 6, 12, and 24 months of age. Data were recorded from these forms into the project’s database by site research coordinators. A Web-based application was used to support data collection for this study. Study staff worked with each site’s clinical informatics team to receive electronic extracts for the clinical database by using a standardized data dictionary of discrete data elements. The study coordinating site provided frequent support to the sites and received extracts from all 4 sites. This was accomplished through biannual extracts from each site via secure pathways. For analyses, the bioinformatics team at the coordinating site created final data reports that were provided to the biostatistics team, which worked closely with the study principal investigators to analyze and interpret the data. The characteristics of the study participants were reported in terms of frequency and percentage, and comparisons between study sites were made using the chi-square test. The prevalence of FA and its CI were calculated by using the exact Clopper-Pearson method. A logistic regression model was used to assess the association of FA with potential risk factors, adjusting for recruitment site and the child’s sex. The analyses were conducted using Stata software, version 14 (Stata Corp, College Station, Tex), with the significance level set at *P* less than .05.

The study was approved and monitored by the central research center (Digestive Disease Research Institute) of the Tehran University ethics committee. Furthermore, the ethics committees of all 4 sites (at the respective host universities) reviewed and approved this protocol. All study participants (parents and guardians) signed an informed consent form.

## Results

### Demographics and early-life characteristics

There were 1220 infants enrolled in this ongoing study; 1055 of these children were followed for 6 months, 812 have been followed for 12 months, and 573 have been followed for 24 months. The expected and actual follow-up rates are detailed in Table E1 (see the Online Repository at www.jaci-global.org). The 4 study sites had similar attrition rates. Gestational age and sex distribution were similar among study sites; however, the rate of premature birth (11.4%) was higher in Semnan than at the other sites (Table I). Maternal education level and age at delivery were also both significantly higher in Semnan than at the other 3 sites. Mode of delivery varied among the study sites, with cesarean section accounting for 53.8% of total deliveries. The rates of cesarean section were higher in Semnan (63.2%) and Rafsanjani (66.2%) than in Yazd (42.3%). Frequency of a family history of AD in a first-degree relative also differed among the 4 sites, with the lowest rate of family history of AD in Isfahan (21.8%) and the highest rate in Yazd (58%) (Table I).

**Table I:** Demographics and early-life characteristics of the study participants in the PERSIAN cohort across 4 study sites

Patient/maternal factors	Yazd	Isfahan	Semnan	Rafsanjan	Total	*P* value
Group size (no.)	338	349	114	254	1055	
Sex, no. (%)						
Male	162 (47.9)	183 (52.4)	64 (56.1)	133 (52.4)	542 (51.4)	.407
Gestational age (wk), no. (%)						
<37	16 (4.7)	11 (3.2)	13 (11.4)	10 (4.1)	50 (4.8)	.004
Cesarean section delivery, no. (%)						
Yes	143 (42.3)	188 (54)	72 (63.2)	147 (66.2)	550 (53.8)	<.001
Ever breast-fed (n = 897), no. (%)						
Yes	290 (89.2)	338 (98)	105 (99.1)	115 (95)	848 (94.5)	<.001
Exclusively breast-fed (n = 895), no. (%)						
Yes	174 (53.9)	249 (72.2)	47 (44.3)	113 (93.4)	583 (65.1)	<.001
Maternal age at delivery (y), no. (%)						.075
<25	75 (22.2)	77 (22.1)	9 (7.9)	48 (21.6)	209 (20.5)	
25-29	92 (27.2)	92 (26.4)	41 (36)	62 (27.9)	287 (28.1)	
30-34	97 (28.7)	113 (32.5)	38 (33.3)	72 (32.4)	320 (31.3)	
≥35	74 (21.9)	66 (19)	26 (22.8)	40 (18)	206 (20.2)	
Maternal educational level, no. (%)						<.001
High school	202 (59.8)	221 (63.5)	41 (36)	106 (51.5)	570 (56.7)	
University degree	121 (35.8)	120 (34.5)	62 (54.4)	90 (43.7)	393 (39.1)	
Postgraduate	15 (4.4)	7 (2)	11 (9.7)	10 (4.9)	43 (4.3)	
Family history of atopy, no. (%)						
Yes	196 (58)	76 (21.8)	44 (38.6)	110 (43.3)	426 (40.4)	<.001

### Cumulative incidence of FA

The cumulative rates of parent-reported FA in the entire cohort were 5.5%, 13.6%, and 18.4% in children younger than 6, 12, and 24 months, respectively. The cumulative rates for pediatrician-diagnosed FA were 2.1%, 5.5%, and 7.7% in children younger than 6, 12, and 24 months, respectively. The cumulative rates of parent-reported and pediatrician-diagnosed FA in all age groups were highest in Yazd. Table II details these rates by age and study site.

**Table II:** Prevalence (95% CI) of parent-reported and pediatrician-diagnosed FA in the PERSIAN cohort across the 4 study sites

Group	Total	Yazd	Isfahan	Semnan	Rafsanjan
Parent-reported FA					
In child younger than 6 mo	5.5 (4.3-7)	9.8 (7-13.4)	2 (1-4.2)	3.5 (1.3-9.1)	5.5 (3.3-9.1)
In child younger than 12 mo	13.6 (11.4-16.2)	18.6 (14.7-23.3)	8.4 (5.6-12.4)	5 (2.1-11.6)	19.2 (13-27.3)
In child younger than 24 mo	18.4 (15.5-21.8)	24.4 (20-29.4)	10.9 (7.6-15.4)		
Pediatrician-diagnosed FA					
In child younger than 6 mo	2.1 (1.4-3.2)	2.7 (1.4-5.2)	1.5 (0.6-3.6)	1.8 (0.4-6.9)	2.4 (1.1-5.2)
In child younger than 12 mo	5.5 (4.1-7.3)	7.5 (5-10.9)	3.3 (1.7-6.2)	3 (1-9.1)	7.5 (3.9-13.9)
In child younger than 24 mo	7.7 (5.7-10.1)	9.9 (7.1-13.7)	4.8 (2.7-8.3)		

In children younger than 6 months, cow’s milk was the most commonly reported food allergen (in 35% of reported cases). This percentage decreased as the children’s age increased (accounting for 20% of all reported FAs in children aged 6 to 12 months and 7.5% of all reported FAs in children aged 24 months). Conversely, reported allergies to fruits and vegetables increased with the advancing age of the children.

### Association of FA with demographics and early-life characteristics

The relationship between demographic and early-life characteristics and the development of FA during the first year of a child’s life is detailed in Table III. Breast-feeding was associated with a decreased cumulative risk of FA at age 12 months, with only 5% of breast-fed children developing pediatrician-diagnosed FA versus 13% of infants who never received breast milk after birth. Exclusive breast-feeding and mode of delivery were not associated with FA risk by the age of 12 months in this study. Maternal age and education also showed no association with FA risk, as was also the case for sex of children and their gestational age. However, having a family history of AD significantly increased the risk of FA, with odds ratios (95% CIs) of 3.14 (2.1-4.8) and 2.57 (1.4-4.8) for parent-reported and pediatrician-diagnosed FA, respectively.

**Table III:** Association of demographics and early-life characteristics of the study participants with cumulative FA rates at the age of 12 months in the PERSIAN cohort study

Patient/maternal factors	Parent-reported FA			Pediatrician-diagnosed FA		
	Prevalence	OR (95% CI)	*P* value	Prevalence	OR (95% CI)	*P* value
Sex						
Male	13.22	Ref.		5.74	Ref.	
Female	14.01	1.07 (0.7-1.6)	.748	5.31	0.92 (0.5-1.7)	.788
Gestational age (wk)						
≥37	13.75	Ref.		5.53	Ref.	
<37	11.43	0.82 (0.3-2.4)	.714	5.71	1.05 (0.2-4.5)	.951
Cesarean section delivery						
No	14.43	Ref.		6.08	Ref.	
Yes	12.86	0.91 (0.6-1.4)	.634	5.00	0.83 (0.5-1.5)	.560
Ever breast-fed						
No	28.26	Ref.		13.04	Ref.	
Yes	12.96	0.39 (0.2-0.8)	.007	5.16	0.37 (0.1-0.9)	.036
Exclusively breast-fed						
No	16.14	Ref.		6.67	Ref.	
Yes	12.43	0.76 (0.5-1.2)	.204	5.05	0.76 (0.4-1.4)	.399
Maternal age at delivery (y)						
<25	12.96	Ref.		5.29	Ref.	
25-29	16.96	1.40 (0.8-2.5)	.247	8.26	1.63 (0.7-3.7)	.241
30-34	12.74	1.00 (0.6-1.8)	.999	4.63	0.88 (0.4-2.1)	.781
≥35	10.90	0.82 (0.4-1.6)	.576	3.21	0.59 (0.2-1.8)	.360
Maternal educational level						
High school	11.97	Ref.		3.85	Ref.	
University degree	12.56	1.21 (0.8-2.2)	.262	5.44	1.73 (1.0-3.4)	.071
Postgraduate	8.8	0.73 (0.2-2.5)	.605	2.94	0.77 (0.1-5.9)	.802
Family history of atopy						
No	7.89	Ref.		3.41	Ref.	
Yes	21.39	3.14 (2.1-4.8)	<.001	8.38	2.57 (1.4-4.8)	.003

*OR,* Odds ratio; *Ref.,* reference group.

∗ORs were adjusted for study sites.

### Associations of AD and FA

It was observed that children diagnosed with FA were significantly more likely to have AD. In the entire cohort, 30.4% of children experienced AD at some point. Active AD at any given age was assessed in association with parent-reported and physician-diagnosed FA at that specific time point (as shown in Table IV).

**Table IV:** Association of AD with FA in different age groups in the PERSIAN cohort study

Group	Parent-reported FA			Pediatrician-diagnosed FA		
	Prevalence (%)	OR∗ (95% CI)	*P* value	Prevalence (%)	OR∗ (95% CI)	*P* value
AD at age <6 mo						
No	4.16			1.06		
Yes	11.58	3.02 (1.7-5.3)	<.001	7.22	7.26 (3.1-17.3)	<.001
AD at age 12 mo						
No	9.05			2.26		
Yes	28.35	3.98 (2.6-6.0)	<.001	15.98	8.22 (4.3-15.8)	<.001
AD at age 24 mo						
No	10.10			2.27		
Yes	32.18	4.22 (2.7-6.7)	<.001	17.24	8.96 (4.2-19.3)	<.001
AD phenotype						
Never/infrequent	10.10			2.27		
Early transient	28.71	3.58 (2.1-6.2)	<.001	11.34	5.5 (2.2-13.7)	<.001
Early persistent	43.08	6.74 (3.7-12.2)	<.001	26.56	15.55 (6.6-36.8)	<.001
Late	43.75	6.92 (2.4-19.6)	<.001	31.25	19.54 (5.6-67.9)	<.001

*OR,* Odds ratio; *Ref.,* reference group.

∗ ORs were adjusted for study sites.

Children with AD were categorized into 4 groups based on timing of the onset and persistence of AD, as detailed in the Methods section. Among the 573 children who completed a 2-year follow-up, early transient AD was reported in 94 children (16.3%), early persistent AD was reported in 64 children (11.1%), and late-onset AD was reported in only 5 children (2.8%). All 3 AD phenotypes were associated with an increased risk of FA compared with the risk in children with no history of AD. Notably, the odds of FA were particularly high in children with persistent AD who had developed their AD before age 1 year and continued to experience active symptoms beyond that age.

## Discussion

To our knowledge, the PERSIAN study is the first birth cohort study reporting the incidence of reported FA in the Middle East. This study provides valuable insight into the incidence of reported FA and AD in various urban areas of a country with historically very low rates of FA that has not yet been studied. The overall rates of physician-diagnosed and parent-reported FA in this cohort in the first 2 years of life were 2% to 7.7% and 5.5% to 18.4%, respectively. The incidences of parent-reported and physician-diagnosed FA in this study are higher than anticipated for the population studied. It is noteworthy that the rate of allergy to fruits and vegetables comprised the majority of parent-reported FAs at age 24 months, which might indicate overreporting of intolerances or oral allergy syndrome as FA. Recent metanalyses provide updated estimates of the prevalence and trends regarding FA in Europe, including studies published between 2012 and 2021. These data showed the rate of FA being 1.7% to 28.5% in infants younger than 1 year and 1.6% to 38.2% in children aged 2 to 5 years. The reported rates in our cohort were within these ranges.^10^^,^^11^ A large survey-based study aimed to understand the global patterns and prevalence of FA by surveying several national member societies of the World Allergy Organization, including 10 in Middle Eastern countries.^4^ This article reported a global increase in the prevalence of FA.^4^ The limited available data from the Middle East region are mostly parent reported, with FA rates near 7% to 17% in children older than 5 years; however, the data in younger children from this region are even more limited.^4^^,^^12^ Similar to many other countries in the region, Iran is still considered an economically emerging country. It has undergone rapid modern urbanization over the past few decades, suggesting that urbanization may play a role in development of FA in Iran. Interestingly, there were also differences in FA prevalence within the different study sites in Iran. The highest prevalence of FA was in children residing in Yazd, which is the most urban city of the 4 study sites, with a dense inner-city population. The city planning strategy and urbanization of Yazd mimics that of Western countries.^13^ These findings are consistent with those of studies in the United States, which have found that children living in inner cities are at increased risk for the development of FA and other allergic conditions.

There are many other factors as well that could explain this pattern of atopic disease in a previously nonendemic area. Some of these factors include globalization and universal changes in diet and environment. Diet has been shown to play a key role in the infant gut microbiome and development of atopy later in life. There are key differences in diet around the globe, with the Western diet typically consisting of higher amounts of refined sugars and lesser amounts fresh fruits and vegetables, both of which are linked to AD and FA.^14^, ^15^, ^16^ Previous studies have found that when individuals move away from a natural diet (such as a diet containing fresh fruits, vegetables, proteins, and less refined sugar), the gut microbiome lacks many of the gut microbiota that are protective against development of atopy.^5^^,^^16^ Additionally, urbanization is associated with decreased exposure to the natural environment, which is essential for a healthy and protective gut microbiome.

In this cohort, we did not observe any significant association of exclusive of breast-feeding with FA; however, any breast-feeding in early life was found to be protective against the development of FA. Prior studies have found conflicting results regarding the role of breast-feeding in preventing FA. Some studies suggest that breast-feeding may be protective against the development of FA owing to the transfer of protective specific IgA to infants, which may prevent allergic sensitization and avoidance of early exposure to potential food allergens.^16^^,^^17^ However, the most recent consensus guidelines from the American Academy of Allergy, Asthma & Immunology advise that although breast-feeding has many benefits for nutrition and infectious immunity, there is no conclusive evidence that it can prevent or delay FA.^18^ Additional studies are needed to further investigate the role of breast-feeding and the impact of maternal diet on FA.

Our study did not find any significant association between development of FA and mode of delivery, maternal education, or maternal age. Although a Swedish cohort study did find a possible association between delivery via cesarean section and FA, our data are consistent with data from the HealthNuts study from Australia, which also found no significant association between mode of delivery and development of FA.^18^ The differential results might be due to other factors associated with elective versus indicated or urgent cesarean sections across these studies. In Iran, many healthy expectant mothers chose cesarean section despite not having a medical indication for the procedure, which is reflective of the high rate of cesarean section in this cohort (53.8%),^19^^,^^20^ whereas in Sweden cesarean section is performed because of a medical indication, with national rates of 15% to 17%.^21^

Our study found a significant link between AD and FA. Children with a diagnosis of FA were more likely to have AD. The odds of a child developing FA were highest in those who developed persistent AD before age 1 year. These data are consistent with a birth cohort study from Japan, which also found that early-onset persistent AD was strongly associated with development of FA.^6^ AD has been implicated in the development of FA through many intrinsic and skin-related mechanisms, including patients with AD being at higher risk of introduction of food allergens via an impaired skin barrier.^14^^,^^22^^,^^23^ It is noteworthy that the association between early transient AD and FA was markedly lower than the association between persistent AD and FA (odds ratio = 3.6 vs 6.7). This might suggest that early management of AD in infancy and maintenance of an intact skin barrier could be a potential goal for preventing development of FA later in childhood. However, the stronger link of FA with persistent AD than with transient AD might be due to the differential mechanism of these 2 AD phenotypes and indicative of a more sustained inflammatory response resulting in the development of FA in the group with persistent AD.

Because of the coronavirus disease 2019 (COVID-19) pandemic, the current study had to stop recruitment in early 2020 and was closed with the reported recruitment of 1220 newborns. Nonetheless, the cohort captured 5.7% of the expected births within the target populations during the period of the study. Furthermore, many of the children who were recruited right before the pandemic were not fully followed up. Some of the sites were closed for a few months as resources were redirected to urgent health care needs. Additionally, there was significant hesitation among parents to visit any medical facility in 2020-2021, which resulted in many children being lost to follow-up. The expected and actual follow-up rates are detailed in Table E1. It is noteworthy that 83% and 72% of expected cases had follow-up data at 12 and 24 months. Although efforts were made to follow up with the entire cohort equally, regardless of any conditions, it is possible that individuals with FA tended to remain in the study longer and were more consistent with follow-ups. This may have led to higher than actual numbers in the observations. Furthermore, the data in this study are based on parents’ reports and interviews and not on confirmation of FA by evidence of sensitization plus history as part of the cohort, which is another limitation. Some other birth cohorts have used measurements of specific IgE and skin testing to confirm FA, which are lacking in our study because of a lack of resources. Overall, the PERSIAN study provides valuable insight into the incidence of reported FA in the Middle East, which has not previously been reported on, and it is crucial to our understanding of global FA prevalence. It is also the first birth cohort study in this region to have been performed using standardized methodologies. Our study demonstrates an incidence of FA in an economically emerging country, which, interestingly, is much higher than predicted and aligns with the incidence of FA being seen in Westernized nations. The PERSIAN study identifies some possible protective factors in preventing the development of FA, such as breast-feeding and early management of AD. Additional future analyses of this birth cohort as it matures are needed to better characterize risk factors and protective factors in the development of FA and other atopic diseases, and they can help shed light on the underlying causes of the observed global trend toward an increasing incidence of FA.

**Clinical implications**

The first birth cohort study examining FA in the Middle East provides valuable insight into prevalence of reported FA in early life and its associated factors in Iran.

## Disclosure Statement

Supported by the National Institute for Medical Research Development in Iran (grant 963809 [Role of Environmental Factors and Diet in Early-Life Gut Microbiome and Atopic Diseases in Iran]).

Disclosure of potential conflict of interest: The authors declare that they have no relevant conflicts of interest.
