# Supplementary material for: Prevalence of food allergy and its association with atopic dermatitis in Iran: Results from the PERSIAN birth cohort
Source: J Allergy Clin Immunol Glob. 2024 Dec 10;4(1):100385. doi: 10.1016/j.jacig.2024.100385 (PMC11742619; doi:10.1016/j.jacig.2024.100385)
Supplement: Supplementary data [file mmc1.docx]

| Supplementary Table 1: Expected and percentage of actual follow up rates | | | | |
| --- | --- | --- | --- | --- |
|  | **Recruited** | **6 months** | **12 months** | **24 months** |
| expected N (% expected retention rate) | 1220 | 1098 (90%) | 976 (80%) | 793(65%) |
| completed, N (% of actual retention) | 1220 | 1055(86.4%) | 812(66.5%) | 573 (46.9%) |
| % of actual/expected number at each time point | n/a | 96% | 83% | 72% |

**Supplementary References**

1. Prescott S, Allen KJ. Food allergy: riding the second wave of the allergy epidemic. Pediatr Allergy Immunol. 2011;22(2):155-60.

2. Bilaver LA, Chadha AS, Doshi P, O'Dwyer L, Gupta RS. Economic burden of food allergy: A systematic review. Ann Allergy Asthma Immunol. 2019;122(4):373-80 e1.

3. Leung ASY, Wong GWK, Tang MLK. Food allergy in the developing world. J Allergy Clin Immunol. 2018;141(1):76-8 e1.

4. Prescott SL, Pawankar R, Allen KJ, Campbell DE, Sinn J, Fiocchi A, et al. A global survey of changing patterns of food allergy burden in children. World Allergy Organ J. 2013;6(1):21.

5. Woods RK, Stoney RM, Raven J, Walters EH, Abramson M, Thien FC. Reported adverse food reactions overestimate true food allergy in the community. Eur J Clin Nutr. 2002;56(1):31-6.

6. Yamamoto-Hanada K, Suzuki Y, Yang L, Saito-Abe M, Sato M, Mezawa H, et al. Persistent eczema leads to both impaired growth and food allergy: JECS birth cohort. PLoS One. 2021;16(12):e0260447.

7. Burney PG, Potts J, Kummeling I, Mills EN, Clausen M, Dubakiene R, et al. The prevalence and distribution of food sensitization in European adults. Allergy. 2014;69(3):365-71.

8. Sicherer SH, Sampson HA. Food allergy: Epidemiology, pathogenesis, diagnosis, and treatment. J Allergy Clin Immunol. 2014;133(2):291-307; quiz 8.

9. Poustchi H, Eghtesad S, Kamangar F, Etemadi A, Keshtkar AA, Hekmatdoost A, et al. Prospective Epidemiological Research Studies in Iran (the PERSIAN Cohort Study): Rationale, Objectives, and Design. Am J Epidemiol. 2018;187(4):647-55.

10. Spolidoro GCI, Ali MM, Amera YT, Nyassi S, Lisik D, Ioannidou A, et al. Prevalence estimates of eight big food allergies in Europe: Updated systematic review and meta-analysis. Allergy. 2023;78(9):2361-417.

11. Spolidoro GCI, Amera YT, Ali MM, Nyassi S, Lisik D, Ioannidou A, et al. Frequency of food allergy in Europe: An updated systematic review and meta-analysis. Allergy. 2023;78(2):351-68.

12. Alzahrani A, Alrebaiee S, Alsalmi S, Althomali M, Alsofyani R, Alkhudaydi F, et al. Prevalence of Parent-Reported Food Allergies and Associated Risk Predictors Among Children in Saudi Arabia. Cureus. 2023;15(1):e33974.

13. Rahbarianyazd R. The emergence and evolution of Yazd City, Iran: From a concentric planned to sectoral model. 2022 [Available from: <https://doi.org/10.1016/j.cities.2022.103592>.

14. Jungles K, Tran TDB, Botha M, Rasmussen HE, Teixeira-Reis V, Sodergren E, et al. Association of gut microbiota and environment in children with AD, comparison of three cohorts of children. Clin Exp Allergy. 2022;52(3):447-50.

15. Dolence JJ, Kita H. Allergic sensitization to peanuts is enhanced in mice fed a high-fat diet. AIMS Allergy Immunol. 2020;4(4):88-99.

16. Fleischer DM, Chan ES, Venter C, Spergel JM, Abrams EM, Stukus D, et al. A Consensus Approach to the Primary Prevention of Food Allergy Through Nutrition: Guidance from the American Academy of Allergy, Asthma, and Immunology; American College of Allergy, Asthma, and Immunology; and the Canadian Society for Allergy and Clinical Immunology. J Allergy Clin Immunol Pract. 2021;9(1):22-43 e4.

17. Wang S, Wei Y, Liu L, Li Z. Association Between Breastmilk Microbiota and Food Allergy in Infants. Front Cell Infect Microbiol. 2021;11:770913.

18. Currell A, Koplin JJ, Lowe AJ, Perrett KP, Ponsonby AL, Tang MLK, et al. Mode of Birth Is Not Associated With Food Allergy Risk in Infants. J Allergy Clin Immunol Pract. 2022;10(8):2135-43 e3.

19. Sadat Z. Reasons for elective cesarean section in Iranian women. Nurs Midwifery Stud. 2014;3(3):e22502.

20. Zakerihamidi M, Latifnejad Roudsari R, Merghati Khoei E. Vaginal Delivery vs. Cesarean Section: A Focused Ethnographic Study of Women's Perceptions in The North of Iran. Int J Community Based Nurs Midwifery. 2015;3(1):39-50.

21. Pyykonen A, Gissler M, Lokkegaard E, Bergholt T, Rasmussen SC, Smarason A, et al. Cesarean section trends in the Nordic Countries - a comparative analysis with the Robson classification. Acta Obstet Gynecol Scand. 2017;96(5):607-16.

22. Papapostolou N, Xepapadaki P, Gregoriou S, Makris M. Atopic Dermatitis and Food Allergy: A Complex Interplay What We Know and What We Would Like to Learn. J Clin Med. 2022;11(14).

23. Davidson WF, Leung DYM, Beck LA, Berin CM, Boguniewicz M, Busse WW, et al. Report from the National Institute of Allergy and Infectious Diseases workshop on "Atopic dermatitis and the atopic march: Mechanisms and interventions". J Allergy Clin Immunol. 2019;143(3):894-913.
